# Nitrogen deposition does not affect the impact of shade on *Quercus acutissima* seedlings

**DOI:** 10.1371/journal.pone.0194261

**Published:** 2018-03-13

**Authors:** Mingyan Li, Weihua Guo, Ning Du, Zhenwei Xu, Xiao Guo

**Affiliations:** 1 Institute of Ecology and Biodiversity, College of Life Sciences, Shandong University, Jinan, Shandong, P.R. China; 2 Shandong Provincial Engineering and Technology Research Center for Vegetation Ecology, Shandong University, Jinan, Shandong, P.R. China; 3 College of Landscape Architecture and Forestry, Qingdao Agricultural University, Qingdao, Shandong, P.R. China; University of Alberta, CANADA

## Abstract

Light and atmospheric nitrogen (N) deposition are among the important environmental factors influencing plant growth and forest regeneration. We used *Quercus acutissima*, a dominant broadleaf tree species native to the deciduous forests of Northern China, to study the combined effects of light exposure and N addition on leaf physiology and individual plant growth. In the greenhouse, we exposed *Quercus acutissima* seedlings to one of two light conditions (8% and 80% of full irradiation) and one of three N treatments (0, 6, and 12 g N m^−2^ y^−1^). After 87 d, we observed that nitrogen deposition had no significant effects on the seedlings regardless of light exposure. In addition, shade significantly reduced plant height, basal diameter, leaf number, total biomass, gas exchange capacity, and carbohydrate content. In contrast, however, shade significantly increased the amount of photosynthetic pigment, above-ground biomass allocation, and specific leaf area. There was also a hierarchical plasticity among the different seedling characteristics. Compared to traits of growth, biomass, biomass allocation and leaf morphology, the leaf physiology, including photosynthetic pigment, gas exchange, carbohydrate, and PUNE, is more sensitive to light conditions. Among the biomass allocation parameters, the leaf and root mass ratios had a relatively low phenotypic plasticity. The seedlings had high foliar physiological plasticity under various light conditions. Nevertheless, we recommend high irradiance to maintain vigorous seedling growth and, in turn, promote the restoration and reconstruction of vegetation.

## Introduction

Light is one of the most important ecological factors affecting plant growth [1, 2]. It influences leaf traits, regulates plant growth, and determines plant survival [3–5]. In natural conditions, however, sunlight must reach the forest floor through the canopy, which limits plant growth and impedes forest regeneration [6].

Nitrogen deposition from fertilizer use and fossil fuel burning has increased substantially since the onset of the Industrial Revolution [7, 8]. In 1860, ~34 Tg N y^−1^ was deposited on the Earth’s surface. By 1995, this rate had increased to ~100 Tg N y^−1^. It is estimated that by 2050, the N deposition rate will further increase to ~200 Tg N y^−1^[9]. Atmospheric nitrogen inputs have enhanced the availability of nitrogen in forest ecosystems [10]. When the N supply is elevated, soil available nitrogen increases and plant production is promoted [11, 12]. Nevertheless, excess N deposition may acidify and cause base cations to leach from the soil [13] which perturbs plant nutrient balances [14]. Therefore, excess N deposition inhibits plant growth and hinders the revegetation process [12, 15].

In natural environments, plants are subjected to a variety of environmental factors like N deposition and low irradiance. In general, soil N levels may be temporarily higher than those of the forest understory [16, 17]. Plant growth under high light intensity is more limited by nutrient restriction than that under low light [16]. Therefore, N addition facilitates plant growth under high light levels [18, 19]. Some studies demonstrated that nitrogen deposition increases the growth of *Larix kaempferi* and *Robinia pseudoacacia* seedlings under high illumination conditions [20, 21]. Nitrogen plays important roles in photosynthesis and the production of light-harvesting pigments and diphosphoribulose carboxylase [22]. In the shade, plants invest more resources in the shoots than the roots in order to capture sunlight more effectively [23, 24]. Nitrogen deposition increases shoot size [18, 25]. In the shade, therefore, N deposition can help plants elongate shoots and obtain more solar energy. Moreover, some studies suggest that nutrient availability has a greater impact on plant growth under higher light intensity than it does when light levels are low [18, 19]. Therefore, an interaction may exist between the nutrient- and light levels. Nevertheless, the nature of this interaction may vary among different species [16].

Plants produce both non-structural carbohydrates (NSC, including soluble sugars and starch) and structural carbohydrates (such as cellulose) [26]. These are the partly products of photosynthesis [27]. Non-structural carbohydrates are carbon and energy reserves, and participate in osmoregulation [28, 29]. The latter is important during environmental stress. For example, dehydrated plants accumulate large quantities of soluble sugars and starch in the leaves, which increases respiration rates [30]. Other studies suggest that water-limited plants transfer far more soluble sugar from the leaves to the roots than well-hydrated plants in order to increase root water uptake, alleviate osmotic stress, and maintain plant growth [31]. Furthermore, foliar NSC is affected by various environmental factors like irradiation and nitrogen addition [29, 32–34]. Cellulose plays an important role in leaf growth and development. Most studies regarding cellulose were conducted on various sclerophyllous leaves [35], forbs, and grasses [36, 37]. To the best of our knowledge, however, very few studies have investigated the impact of environmental factors on cellulose [38]. The allocation of carbohydrates between metabolism and growth and development is a key trait of leaves. Nevertheless, there are relatively few published studies addressing this parameter. The changes in carbohydrate accumulation and allocation that occur in plants in response to various shade and N deposition conditions remain unknown.

Phenotypic plasticity adjusts plant morphology and physiology in response to different environmental conditions [39]. Phenotypic plasticity is species-specific [18, 40–42] and its magnitude differs depending on species attributes such as growth rate, age, and allocation patterns [43]. High phenotypic plasticity facilitates the acclimation to changing environmental conditions even under severe stress [44, 45]. The growth and even the survival of a plant are enhanced when it possesses a high degree of plasticity [46]. A hierarchy of plasticity has been reported among different plant traits [40]. Physiological characteristics and whole plant growth have higher plasticity than morphological characteristics in response to drought and shade [41, 45].

*Quercus acutissima* belongs to the Fagaceae family and is the dominant native broadleaf tree species in the deciduous forests of Northern China [47]. It is extensively used for revegetation in warm temperate regions and is subjected to a wide range of light conditions and N deposition rates. The effects of soil moisture, illumination, N deposition rate, and atmospheric CO_2_ on *Q*. *acutissima* seedlings have been reported [15, 48, 49]. To our knowledge, however, no studies have been made on the morphological and physiological responses of *Q*. *acutissima* seedlings to various combinations of N addition rates and shade conditions. To this end, we conducted a greenhouse experiment using two light conditions and three N deposition levels. Our objectives were to determine whether (1) N deposition has a stronger effect on *Q*. *acutissima* seedling growth under high illumination than it does at low light levels; and (2) there is a hierarchy of phenotypic plasticity among the traits of *Q*. *acutissima* seedlings.

## Materials and methods

### Study site

The research was conducted at the Fanggan Ecological Research Station (36°260’N, 117°270’E) of Shandong University, Shandong Province, China. This station is located in a warm temperate zone with a typical monsoon climate. The mean annual temperature and precipitation are ~13 ± 1°C and ~600–800 mm, respectively. Loam and clay loam are the main soil types in the study area [50]. The seedlings were shielded from precipitation by conducting the entire study in a greenhouse. The facility was ventilated by rolling up the plastic film on the sides. The average N deposition in Northern China ranges from ~1.5–5 g m^-2^ y^-1^ and shows an upward trend [51].

### Plant material

Seeds were collected in September 2015 from the mountains near the research station and stored at 0–4°C over the winter. In March 2016, the seeds were germinated by soaking them in water for 24 h. When their radicles were ~2 cm long, the seedlings were transplanted to plastic pots 25 cm in height × 24 cm in diameter. One seedling was placed in each pot. The substrate consisted of a well-blended mixture of 4.9 kg loam and 1.6 kg sand which was sieved to remove debris and stones. The substrate had a pH of 5.7, 71.40 mg kg^-1^ available N, and 17.38 mg kg^-1^ available P.

### Experimental design

The seedlings were arranged in three different N deposition treatments: 0 (control, N0), 6 g N m^−2^ y^−1^ (N1), and 12 g N m^−2^ y^−1^ (N2). N1 corresponded to the N deposition rates recorded in certain regions of China. N2 represented a projected high N deposition level [15, 52, 53]. According to the report that the ratio of NH4-N and NO3-N in atmospheric N deposition in China was about two in recent years [54], nitrogen deposition was simulated by adding an aqueous ammonium nitrate (NH_4_NO_3_) solution directly to the soil every 2 wks. These solutions were prepared with deionized water. The amount of NH_4_NO_3_ used was calculated on the basis of the target N addition levels.

Each N deposition level was exposed to one of two light regimes: 8% of full radiation (low light, L1), and 80% of full radiation (high light, L2). L1 corresponded to the understory in deciduous broadleaf forests and was simulated with a woven black nylon net shelter placed over the seedlings in the greenhouse. L2 represented the canopy gap light condition and was reproduced with the plastic film over the greenhouse [24]. The experimental design was full factorial with six treatments in total. Each treatment consisted of five replicates, and each pot was one replicate. The 30 pots were arranged randomly and rearranged regularly during the experiment. The study was conducted over the entire growing season from June 20 to September 12, 2016. Every pot received compensatory water by weighing daily at 18:00 to maintain the 65%±5% of the field capacity amount different treatments, and insects and weeds were manually controlled.

### Measurements

Seedling height, basal diameter, crown area, and leaf number were recorded at the end of the experiment. Five seedlings were measured per treatment. Crown area = crown width × crown length × 0.25π.

The maximum net photosynthetic rate (A_max_), transpiration rate (E), and stomatal conductance (Gs) were measured between 09h00 and 11h30 on August 27, 2016 using a portable leaf gas exchange system (GFS-3000, Walz GmbH, Effeltrich, Germany). A saturated photosynthetic photon flux density of 1,200 μmol m^-2^ s^-1^ was determined by pre-experimentation and provided by an external red-blue light. The expanded mature leaf of each upper shoot from three seedlings was selected for every treatment. During the measurements, the CO_2_ concentration, mean ambient temperature, and relative humidity in the leaf cuvette were 400 μmol mol^-1^, 28°C, and 70%, respectively.

Leaf morphology was examined at the end of the experiment. Five fully developed apical leaves from each treatment were scanned. After the images were captured, the thicknesses of the five leaves were measured using a micrometer caliper. The leaves were then oven-dried at 80°C for 48 h and their dry weights were determined. The leaf area was calculated with WinFOLIA Pro 2009a (Regent Instruments, Inc., Quebec, QC, Canada). The specific leaf area (SLA) was calculated as the leaf area divided by the leaf dry mass.

Photosynthetic pigment content was determined using a 722S visible light spectrophotometer (Leng Guang, Inc., Shanghai, China). The chlorophyll a, (Chl a), chlorophyll b (Chl b), and carotenoid (Car) of the five leaves from each treatment were extracted in 95% v/v ethanol according to the methods described in [55]. Chl a, Chl b, Car, total chlorophyll (Chl a + Chl b), and the Chl a/Chl b were calculated on a fresh weight basis.

Leaves of four seedlings from each treatment were selected to measure foliar soluble sugars (SS), starch (St), and cellulose (C). The leaves were dried at 80°C for 48 h, weighed, and pulverized. Dried leaf tissue (50 mg) was prepared for SS extraction by macerating it in 80% v/v ethanol in a water bath [56]. The digest was centrifuged and the supernatant was decanted and used for SS determination. The residue was digested to glucose with perchloric acid to determine St levels. Samples from which the SS and St had been extracted were combined with excess acid and alkali to remove impurities. The residues were washed with deionized water, dried at 80°C for 12 h, and weighed. SS and St were determined by phenol–sulfuric acid colorimetric assay [57] and recorded on a dry mass basis. Foliar SS, St, and CF were calculated relative to leaf biomass and recorded as w/w concentrations (mg g^-1^). The ratios of soluble sugar, starch, and cellulose were calculated as follows:

Solublesugarratio=solublesugar/(solublesugar+starch+cellulose)

Starchratio=starch/(solublesugar+starch+cellulose)

Celluloseratio=cellulose/(solublesugar+starch+cellulose)

At the end of the experiment, five seedlings from each treatment were dissected into three organs (root, stem, and leaf). Each organ was weighed after oven-drying at 80°C for 48 h. Total biomass was calculated by the addition of all the plant parts. Specific plant part ratio was calculated as its mass divided by the total plant mass. Root to shoot ratio was calculated as root plant parts divided by shoot plant parts.

After biomass determination, four or five fully expanded leaves were selected from each treatment to determine leaf nitrogen (LN) concentrations by the Kjeldahl method [15]. Photosynthetic N-use efficiency (PNUE) was calculated as follows [58]:

$$PNUE=A_{max}/\left(LN\;\times \;LMA\;\times \;1/14\right)$$

where LMA is the leaf mass per area and 14 is the relative atomic mass of N.

### Statistical analysis

Two-way analysis of variance (ANOVA) was run to find the differences among the light regimes, the N deposition rates, and their interactions for every parameter of *Q*. *acutissima*. One-way ANOVA and Duncan’s multiple range tests (DMRT) were applied to detect the differences among treatments. The data were first checked for normality and homogeneity of variance using Levene’s test. When and where necessary, the data were log-transformed to meet the assumptions of normality and homogeneity. All statistical analyses were performed with SPSS v. 22.0 (SPSS Inc., Chicago, IL, USA). Figures were drawn using Origin v. 9.0 (OriginLab Corp., Northampton, MA, USA). The plasticity of the traits among the treatments was calculated as the difference between the maximum and minimum values divided by the maximum treatment value.

## Results

**Fig 1:**
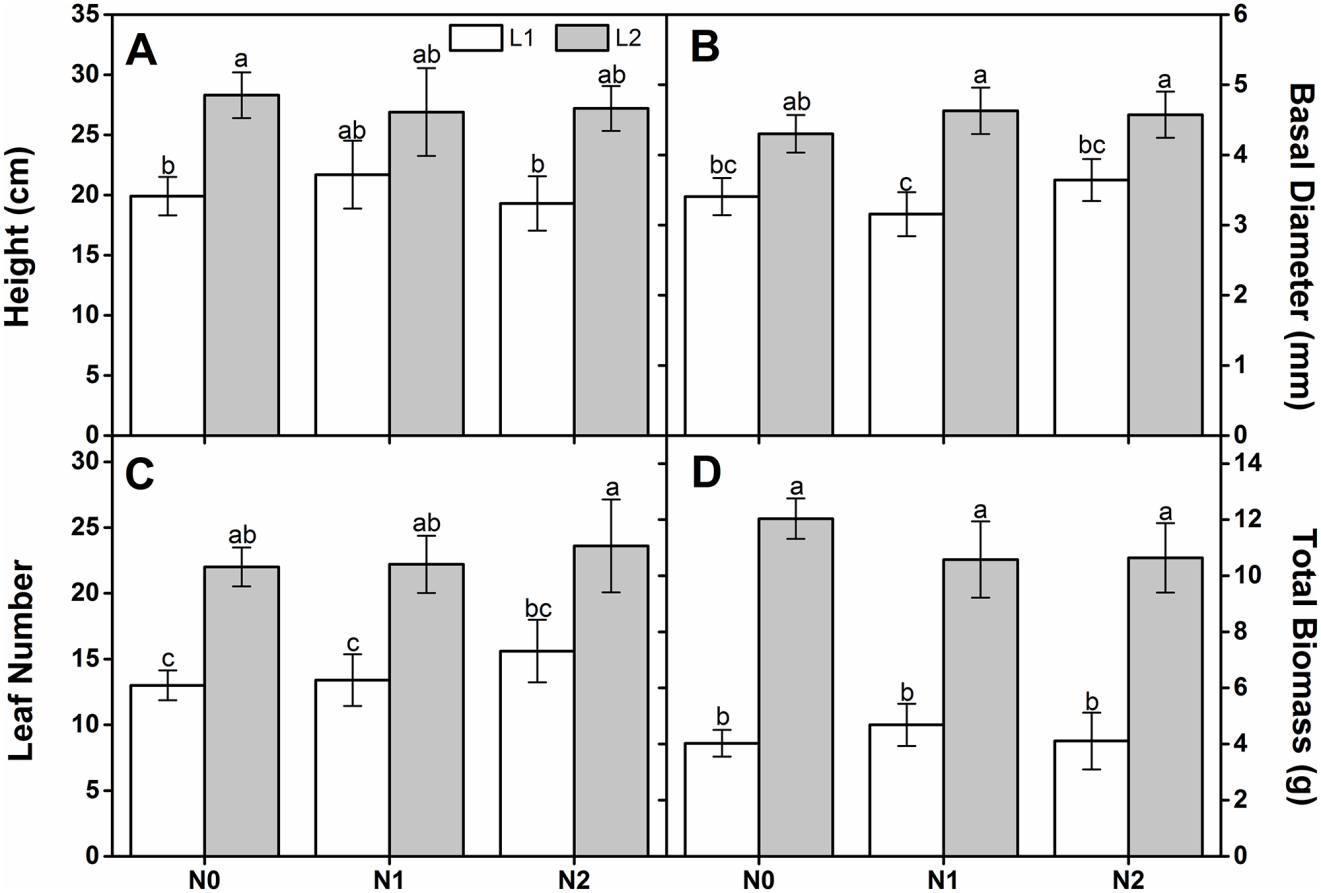
Final height (A), basal diameter(B), leaf number(C) and total biomass (D) for one-year-old *Q*. *acutissima* seedlings subjected to different light- and N treatments (mean ± standard error (SE), *n* = 5). Different letters within the same column denote significant differences at *p* < 0.05 according to Duncan’s test.

Light regime significantly affected the height, basal diameter, leaf number, and total biomass. N deposition rate and its interaction with light regime had no significant effect on any growth or biomass parameters (Table 1). Seedling height under L2 was significantly higher than that under L1 in the absence of N deposition (Fig 1A). Nevertheless, the basal diameter under L1 was lower than that under L2 in the presence of N deposition (Fig 1B). Leaf number and total biomass significantly increased with light intensity under all N deposition rates (Fig 1C and 1D).

**Table 1:** Final two-way ANOVA of different treatments on *Q*. *acutissima*.

Parameters	Light		Nitrogen		Light × Nitrogen	
	*F*	*P*	*F*	*P*	*F*	*P*
Height (cm)	12.821	**0.002**	0.103	0.902	0.247	0.783
Basal Diameter (mm)	19.890	**0.001**	0.414	0.666	0.576	0.569
Leaf Number	22.026	**0.001**	0.512	0.606	0.028	0.973
Total Biomass (g)	73.072	**0.001**	0.230	0.796	0.618	0.547
LMR	9.337	**0.005**	0.093	0.911	0.035	0.965
SMR	5.188	**0.032**	0.419	0.662	0.930	0.408
RMR	11.533	**0.002**	0.296	0.746	0.498	0.614
R/S	12.966	**0.001**	0.261	0.773	0.194	0.825
Chlorophyll a (mg g^-1^)	48.089	**0.001**	2.244	0.128	2.623	0.093
Chlorophyll b (mg g^-1^)	58.158	**0.001**	1.507	0.242	1.686	0.206
Carotenoids (mg g^-1^)	22.988	**0.001**	1.820	0.184	0.579	0.568
Total Chlorophyll (mg g^-1^)	54.370	**0.001**	2.059	0.150	2.372	0.115
Chlorophyll a/b	14.491	**0.001**	0.360	0.702	1.510	0.241
Soluble Sugar (mg g^-1^)	18.089	**0.001**	0.130	0.879	0.307	0.738
Starch (mg g^-1^)	24.449	**0.001**	0.302	0.742	0.169	0.845
Cellulose (g g^-1^)	8.459	**0.008**	0.159	0.854	1.290	0.294
Soluble Sugar ratio	0.015	0.902	1.137	0.337	1.128	0.340
Starch ratio	12.204	**0.002**	0.236	0.792	0.204	0.817
Cellulose ratio	10.119	**0.004**	0.266	0.769	0.572	0.572
A_max_ (μmol m^−2^ s^−1^)	16.059	**0.002**	0.233	0.796	0.003	0.997
E (mmol m^−2^ s^−1^)	25.588	**0.001**	1.968	0.182	0.257	0.778
Gs (mmol m^−2^ s^−1^)	47.082	**0.001**	1.775	0.211	2.502	0.124
LN (mg g^-1^)	12.150	**0.002**	0.540	0.590	0.501	0.612
PNUE (μmol mol^-1^ N s^-1^)	8.734	**0.012**	3.162	0.079	0.490	0.624
Leaf Thickness (mm)	34.355	**0.001**	0.512	0.606	0.933	0.407
SLA (cm^2^ g^-1^)	14.084	**0.001**	1.206	0.317	0.502	0.611

Numbers in bold are significant at *p* < 0.05.

LMR, leaf mass ratio; SMR, stem mass ratio; RMR, root mass ratio; R/S, root to shoot mass ratio; A_max_, light-saturated photosynthetic rate; E, transpiration rate; Gs, stomatal conductance; iWUE, intrinsic water-use efficiency; LN, leaf N content; PUNE, photosynthetic nitrogen use efficiency; SLA, specific leaf area.

**Fig 2:**
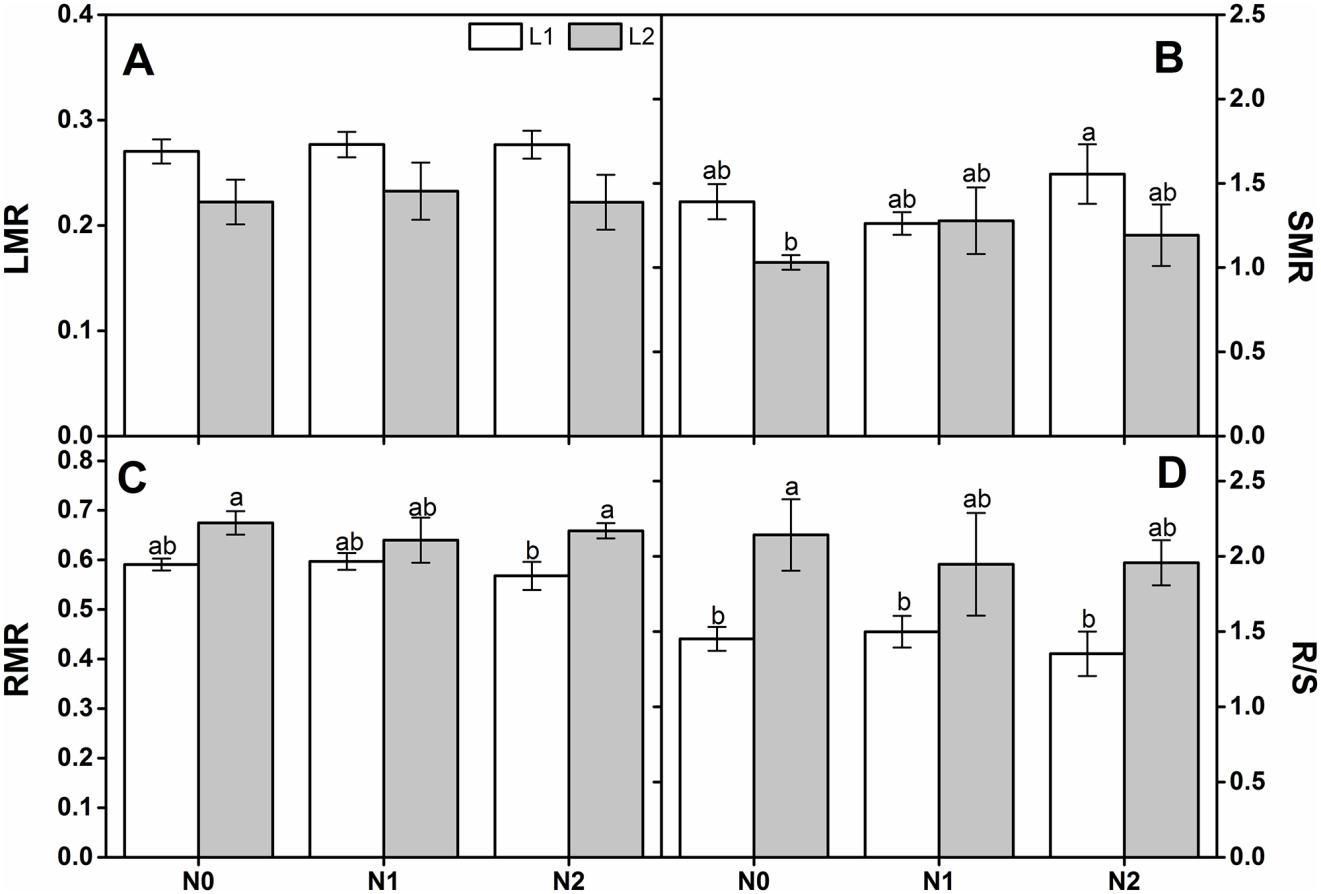
Comparison of final biomass allocation in one-year-old *Q*. *acutissima* seedlings subjected to different light intensities and N treatments (mean ± SE, *n* = 5). Leaf mass ratio (LMR) (A), stem mass ratio (SMR) (B), root mass ratio (RMR) (C) and root/shoot ratio (R/S) (D) of *Q*. *acutissima* seedlings. Different letters within a column denote significant differences at *p* < 0.05 according to Duncan’s test.

N deposition and its interaction with light intensity did not significantly affect LMR, SMR, RMR, or R/S. Light regime significantly affected all biomass allocations (Table 1). Both LMR and SMR increased with light intensity under all N deposition rates (Fig 2A and 2B). Under L2, however, RMR and R/S were higher than they were at L1 (Fig 2C and 2D).

**Fig 3:**
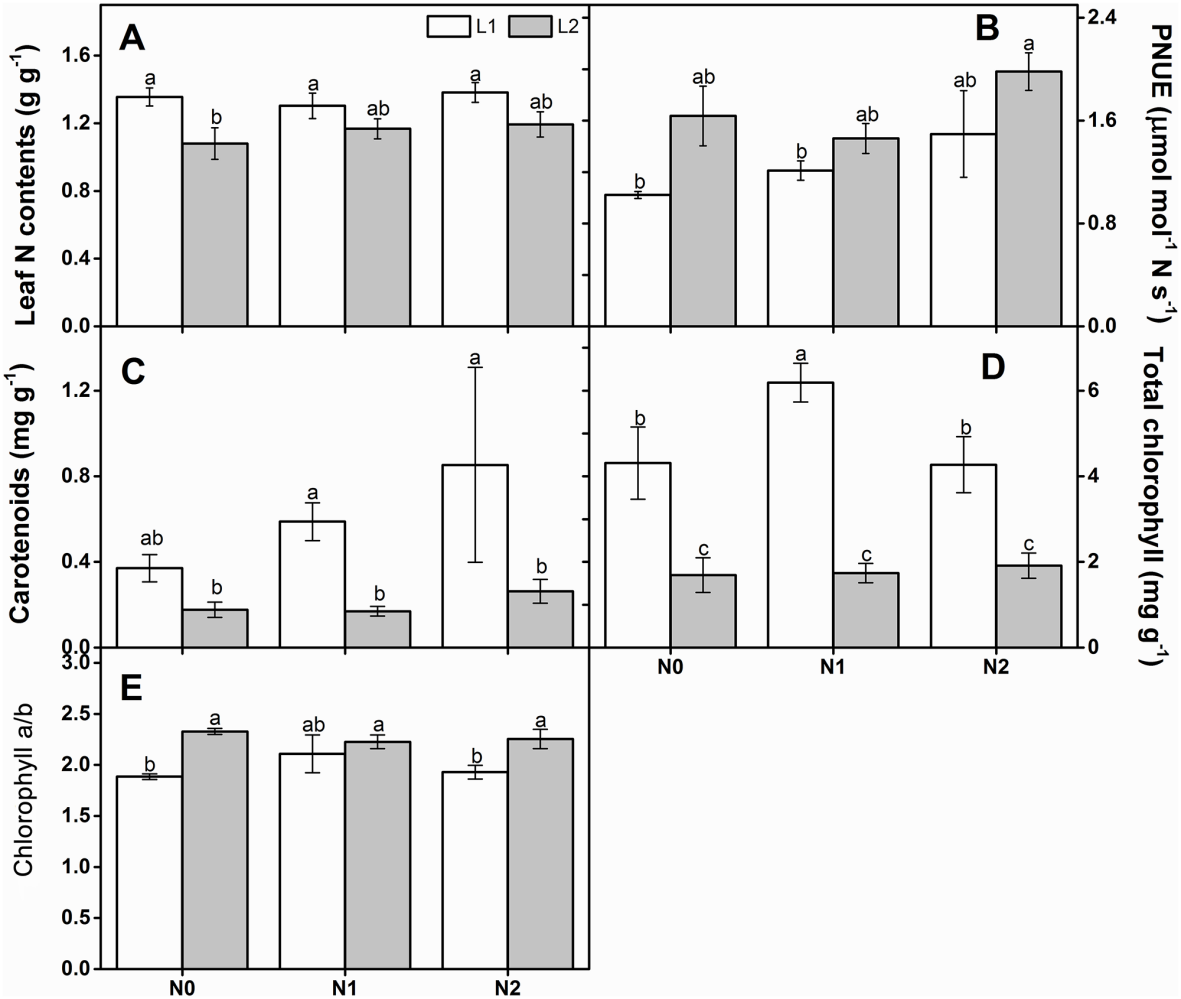
Leaf N content (A), PUNE (B), carotenoids (C), Total chlorophyll (D) and chl a/b (E) of *Q*. *acutissima* seedlings under different light intensities and nitrogen treatments. Bars represent mean ± standard error (SE, n = 5). Values in the same column with different letters are significantly different at *p* < 0.05.

Leaf N content and PNUE were significantly different under the two light regimes according to two-way ANOVA (Table 1). In the N0 treatment, the leaf N content under L2 exposure was significantly lower than that under L1 (Fig 3A). With increasing light intensity, PNUE increased under all N deposition rates (Fig 3B). Photosynthetic pigment levels were apparently affected by light intensity (Table 1). Carotenoids and total chlorophyll were significantly higher under L1 than that they were under L2 (Fig 3C and 3D). Nevertheless, the chl a/b ratio significantly increased with light intensity (Fig 3E).

**Fig 4:**
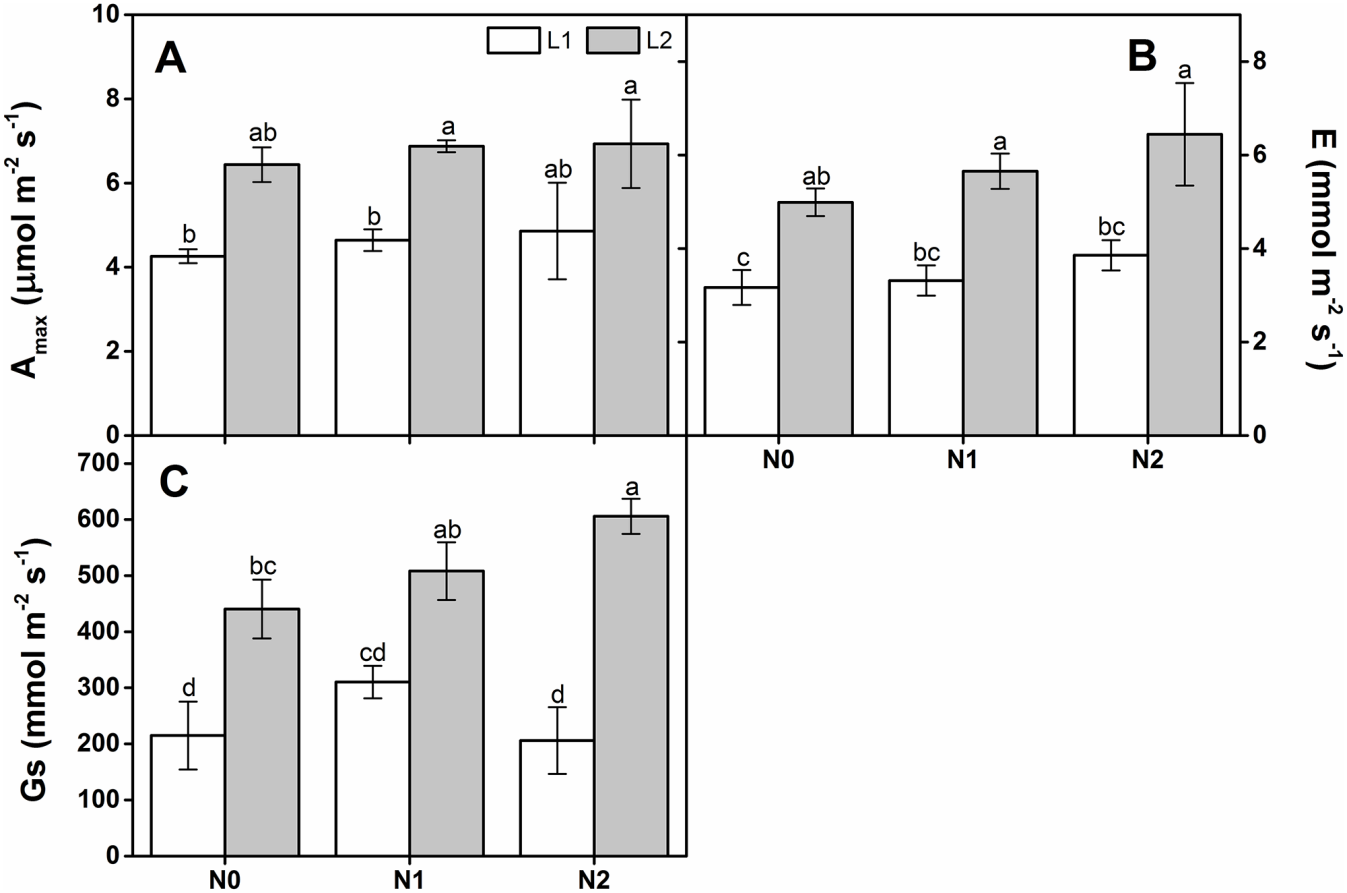
Comparisons of gas exchange rates in one-year-old *Quercus acutissima* seedlings subjected to different light intensities and N treatments (mean ± SE, *n* = 3). A_max_ (A), E (B), and Gs (C). Different letters within the same column denote significant differences at *p* < 0.05 according to Duncan’s test.

A_max_, E, and Gs were significantly affected by light regime. Nevertheless, these parameters did not significantly differ either in response to the N addition rate or its interaction with light intensity (Table 1). The A_max_, E, and Gs of seedlings under L2 were apparently higher than those for seedlings under L1 at all N deposition rates (Fig 4A–4C).

**Fig 5:**
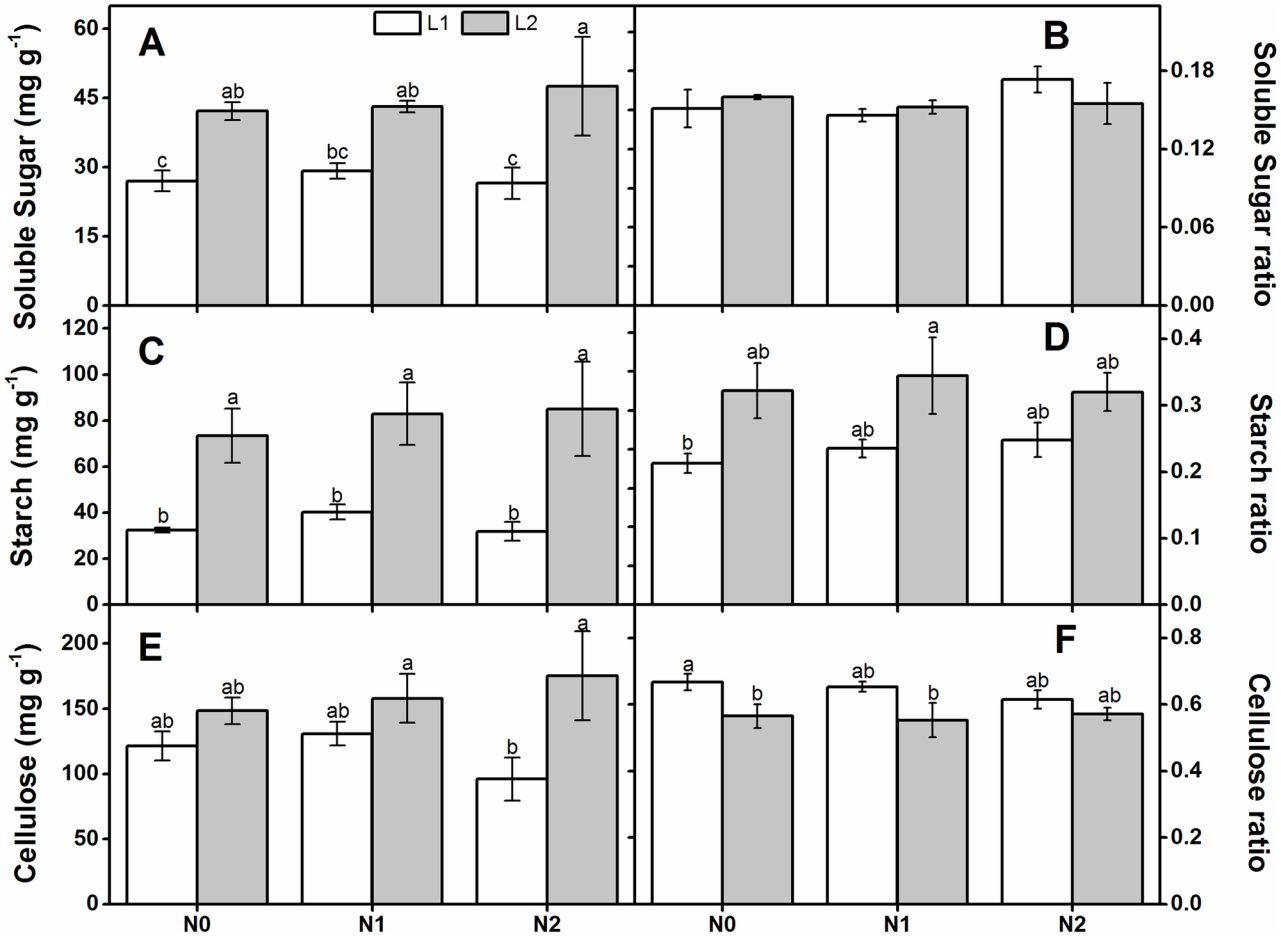
Comparison of carbohydrate accumulation and allocation in one-year-old *Q*. *acutissima* seedlings subjected to different light levels and N treatments (mean ± SE, *n* = 5). Soluble sugar content (A), soluble sugar ratio (B), starch content (C), starch ratio (D), cellulose content (E), and cellulose ratio (F). Different letters within the same column denote significant differences at *p* < 0.05 according to Duncan’s test.

Light level significantly affected leaf carbohydrate content. Neither N deposition rates nor their interaction with light intensity apparently affected leaf carbohydrate content (Table 1). At all N deposition rates, the soluble sugar and starch content under L1were lower than those under L2 (Fig 5A and 5C). In addition, the cellulose content under L2 was significantly higher than that under L1 when the N deposition rate was high (Fig 5E). Both the starch and cellulose ratios in the leaves were affected by light level (Table 1). The starch ratio increased with light level but the cellulose ratio showed the opposite tendency (Fig 5D and 5F).

**Fig 6:**
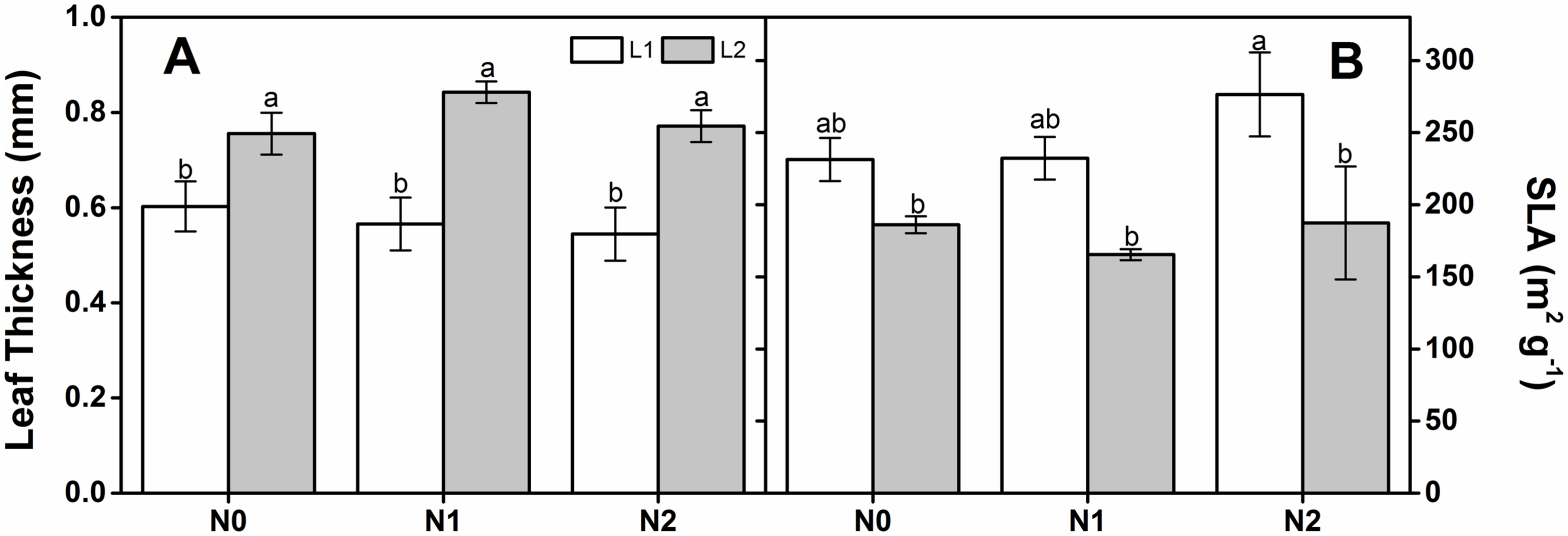
Leaf thickness (A) and SLA (B) of *Q*. *acutissima* seedlings under different light levels and nitrogen treatments. Bars represent mean ± standard error (SE, n = 5). Values accompanied by different letters within the same column are significantly different at *p* < 0.05.

The leaf thickness and SLA were significantly different between the two light regimes according to two-way ANOVA (Table 1). The leaf thickness under L2 was significantly higher than it was under L1 at all N deposition rates (Fig 6A). Nevertheless, SLA decreased with increasing light intensity (Fig 6B).

**Fig 7:**
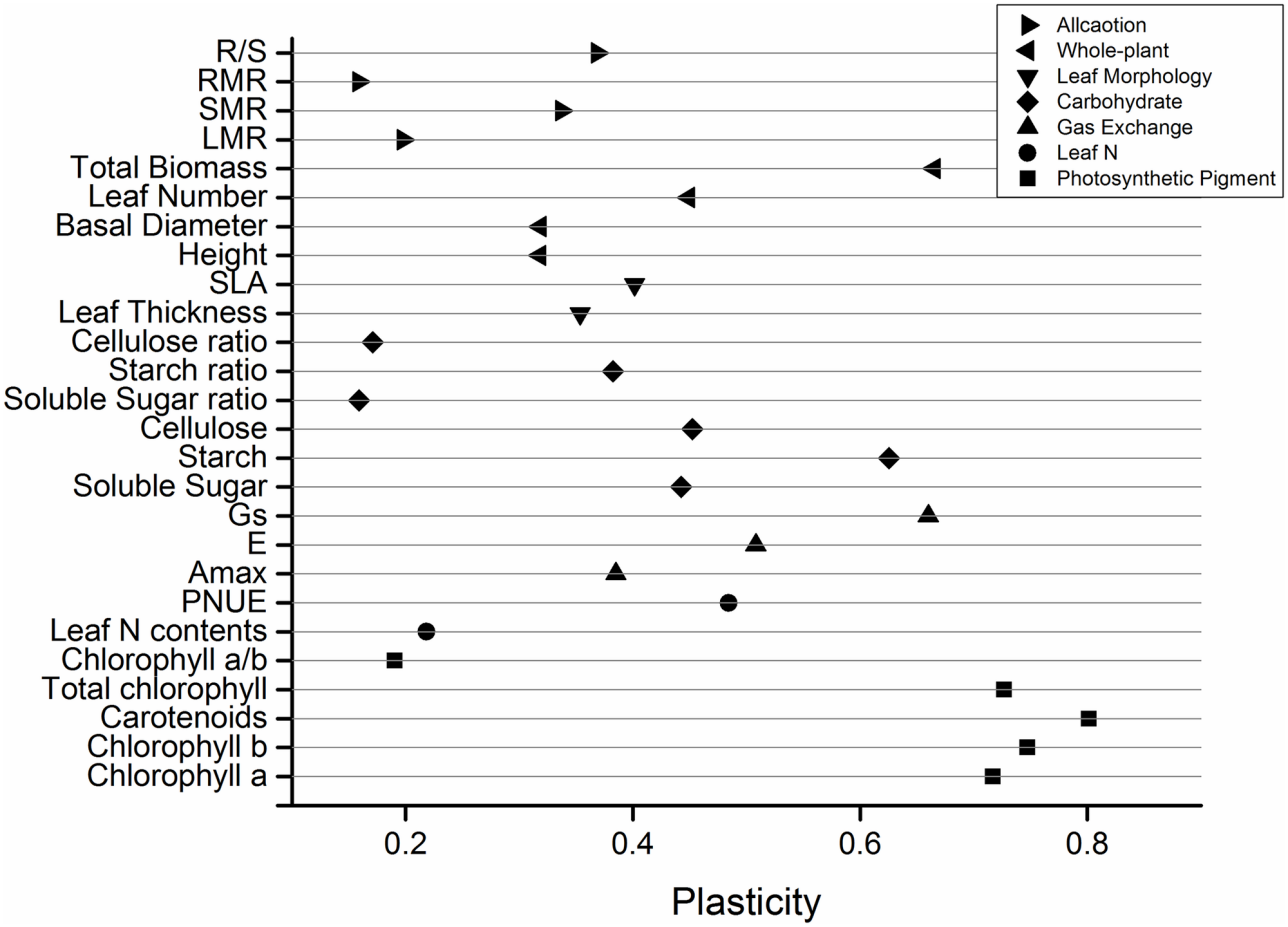
Differences in plasticity of the parameters measured in *Q*. *acutissima* seedlings at different light levels and nitrogen deposition rates. PNUE, photosynthetic N-use efficiency; A_max_, maximum net photosynthetic rate; E, transpiration rate; Gs, stomatal conductance; SLA, specific leaf area; LMR, leaf mass ratio; SMR, stem mass ratio; RMR, root mass ratio; R/S, root to shoot mass ratio.

Plasticity among the different groups varied significantly with light level and N deposition rate (Fig 7). All photosynthetic pigment parameters except for the chl a/b ratio were the most plastic, followed by all of those for carbohydrates except the soluble sugar- and cellulose ratios, then gas exchange, and then leaf morphology. Biomass allocation had the lowest plasticity of all the factors tested.

## Discussion

### Effects of low light on *Q*. *acutissima* seedlings

In this study, seedling height, basal diameter, leaf number, and total biomass were lower in low light levels than that in high light levels. These findings are consistent with those reported in other studies showing that low illumination reduces plant growth rate and biomass accumulation [4, 21, 59]. Interestingly, crown area was not affected by shade conditions. This result contradicts that for *Acer buergerianum* whose crown area was, in fact, negatively influenced by shade [24]. Our results may have been influenced by the tree age [19] and by the specific growth pattern of *Quercus acutissima* whose vertical (acropetal) growth rate exceeds that of its lateral shoots [60]. Previous studies demonstrated that biomass allocation in plants like *Vitex negundo* and *Ziziphus jujuba* were affected by illumination [5]. Our data indicated that *Quercus acutissima* had higher LMR and SMR and lower RMR and R/S in the shade than under high light intensity. When this species is under low irradiance, it may invest more photosynthetic resources in aerial shoot growth and less in root growth. In this way, it can produce more leaves and capture more light energy [20, 61].

Light also affects the physiological parameters of woody plants [41, 62, 63]. In the present study, the mass-based leaf N content decreased with increasing light intensity, which corroborates the findings reported in previous studies [20, 63]. Interestingly, *Quercus acutissima* had the lowest PNUE under low light levels, which means that only a small proportion of the leaf nitrogen was allocated to the photosystem. Carotenoids are biosynthetic chlorophyll precursors [64], which protect the photosynthetic apparatus [65]. In the present study, the carotenoid concentration significantly increased under low light levels. This result aligns with those of previous studies [12, 66]. This phenomenon could be the result of blocking photosynthesis under low light intensity and the accumulation of excess excitation energy. Carotenoid buildup dissipates this energy excess in the form of heat which cannot be utilized for photosynthesis. Therefore, the increase in carotenoid concentration in the shade mitigates damage to the photosystem caused by low-light stress [67]. The total chlorophyll concentration also significantly increased under low light intensity, as reported in previous studies [62, 68]. This result confirmed that plants boost their light-harvesting capacity in order to maintain growth under low light conditions [59]. The chl a/b ratio was higher under the high light regime [69], mainly because a greater investment in chlorophyll b improves PSII function in the shade [24, 69]. A_max_ was at a maximum under high light levels probably because the PNUE and the chl a/b ratio are also elevated in those conditions. This finding was consistent with previous reports indicating that high illumination raised the light saturation point [41, 70] and enhanced the activity of enzymes like Rubisco and ATP synthase [71].

The soluble sugar, starch and cellulose content in the leaves were also influenced by different light regimes. In the present study, all these factors were lower under low light levels than high illumination. The relatively weaker A_max_ in shade conditions may account for this observation [26, 72]. The carbohydrate ratios also responded differently to the two light regimes possibly because the functions of soluble sugar, starch, and cellulose vary with environment [38, 73]. Soluble sugar is osmotically active [28], and adjusts the osmotic potential of the cell fluid in order to maintain plant growth [31]. In this study, the soluble sugar ratio was not affected by different light regimes possibly because the plants were well hydrated and therefore not under osmotic stress. In addition, starch, a carbon reserve, is transformed to soluble sugar to maintain high respiration during plant growth in the shade [72]. For these reasons, the soluble sugar ratio no changed and the starch ratio decreased under low light levels. Interestingly, the cellulose ratio increased under low light intensity. Cellulose is the main cell wall component and varies only slightly with environmental change. However, low light intensity reduced the total structure carbohydrate. Therefore, the cellulose ratio increased under low light condition.

Specific leaf area is a crucial trait that responds to trade-off resource capture and usage [74]. In this study, SLA significantly increased in the shade. This observation is in accordance with those of previous studies [20, 41, 68, 75]. Under high light intensity, leaves tend to increase in thickness to resist overheating. In contrast, the leaves become relatively thin in the shade so they can capture more light [24]. The increase in SLA with decreasing light intensity could be attributed to a homeostatic mechanism that optimizes foliar light capture.

### Effects of N addition on *Q*. *acutissima* seedlings

In this study, the simulated N deposition had relatively little impact on *Q*. *acutissima*. This phenomenon may be correlated with the slow growth rate of this species [60]. Slowly growing plant species have relatively low nutrient demands [76]. In addition, the *Q*. *acutissima* seeds were collected from the mountains in the central region of Shandong province where soil N content is suboptimal for plant growth [77]. Therefore, nitrogen has probably been a limiting nutrient in the local forests for generations. The *Q*. *acutissima* used in our experiment may have adapted to low nitrogen levels and was only weakly responsive to N deposition. The low nitrogen demand of *Q*. *acutissima* and the short experimental duration (~85 d) account for the lack of significant plant responses to N addition. Future research may investigate the effects of long-term nitrogen deposition on slow-growing plants.

### Plastic responses among traits of *Q*. *acutissima* seedlings

In this study, the leaf physiology factors like photosynthetic pigments, PNUE, gas exchange, and carbohydrate content had relatively higher plasticity than the other traits. This observation corroborates those of a previous study demonstrating that the physiological traits of several semi-deciduous shrubs had the highest plasticity in response to environmental changes [45]. In general, leaf physiology is more sensitive to environmental changes than other traits [40, 43, 78]. Therefore, most of the leaf physiology parameters in the present study were highly plastic. Our results also suggest that *Q*. *acutissima* acclimated to the shade mainly by modulating its leaf physiology. Nevertheless, the ratios of chl a/b, soluble sugar, and cellulose had lower degrees of phenotypic plasticity than the other leaf physiological parameters. Therefore, hierarchical plasticity occurs among the various physiological leaf characteristics. This property may be correlated with the functions of these parameters which include the maintenance of normal leaf metabolism and morphology [24, 31].

In the present study, biomass allocation had a lower degree of plasticity than the other functional traits. This finding is consistent with that of another report in which the plasticity of biomass allocation in *Q*. *aliena* seedlings was low in response to variations in light intensity and water availability [41]. This trait may be explained by the fact that the already slow growth rate of the shoots of one-year-old seedlings is further impeded by the shade.

## Conclusions

The present study showed that nitrogen load had no significant effects on *Q*. *acutissima* seedlings regardless of light conditions. Moreover, *Q*. *acutissima* seedlings acclimated to low-light environments. Firstly, the seedlings had higher LMR and SMR and lower RMR and R/S under low light- than high light intensity. This response helps plants capture more light energy. Secondly, the increase in chlorophyll content in shaded leaves enhanced their light-harvesting capacity. Thirdly, the higher SLA at low illumination indicated that the seedlings produced thinner leaves which could capture more solar radiation and maintain growth. Nitrogen deposition had no effect on the seedlings due to their low nutrient demand and the brief duration of this experiment. This study also indicated that a hierarchy of plasticity exists among the different traits of *Q*. *acutissima* seedlings. Most of their physiological parameters were relatively more plastic than the other traits. Therefore, *Q*. *acutissima* acclimated to the shade mainly by regulating leaf physiology. Nevertheless, biomass allocation had low phenotypic plasticity because of the slow growth rate under low light conditions. The growth of the seedlings was inhibited by low illumination despite the relatively high plasticity of their leaf physiology. We therefore recommend high irradiance to maintain the vigorous seedling growth beneficial to the restoration and reconstruction of vegetation. Future research should investigate the long-term effects of light intensity and nitrogen loading on slow-growing species like *Q*. *acutissima*.

## Supporting information

S1 Dataset (XLSX)
